# Assessment of Red Dichromatic Imaging with Indigo Carmine for Identifying Deep Submucosal Invasion in Colorectal Tumors: A Pilot Study

**DOI:** 10.3390/diagnostics16111739

**Published:** 2026-06-05

**Authors:** Toshiki Futakuchi, Naoto Tamai, Mai Fukuda, Yuko Hasegawa, Mamoru Ito, Naoya Tada, Masakuni Kobayashi, Machi Suka, Kazuki Sumiyama

**Affiliations:** 1 Department of Endoscopy, The Jikei University School of Medicine, 3-25-8, Nishi-Shimbashi, Minato-ku, Tokyo 105-8461, Japan; tamai-naoto@jikei.ac.jp (N.T.); maifukuda049@gmail.com (M.F.); yukohasegawa01@gmail.com (Y.H.); naoya6295@gmail.com (N.T.); masakuni1105@me.com (M.K.); kaz_sum@jikei.ac.jp (K.S.); 2 Department of Public Health and Environmental Medicine, The Jikei University School of Medicine, 3-25-8, Nishi-Shimbashi, Minato-ku, Tokyo 105-8461, Japan; suka@jikei.ac.jp

**Keywords:** colorectal tumor, colorectal cancer, colonoscopy, red dichromatic imaging, RDI, invasion depth

## Abstract

**Background/Objectives:** Accurate pT1b diagnosis in colorectal cancer is vital owing to the risk of lymph node metastasis. While Japan NBI (narrow band imaging) Expert Team (JNET) classification is widely applied, accurate diagnosis of type 2B lesions remains challenging, often requiring pit pattern analysis via magnifying chromoendoscopy with crystal violet staining (MCE). However, the clinical application of MCE is limited by potential carcinogenicity and prolonged procedure time. In this study, we aimed to evaluate the diagnostic performance of red dichromatic imaging with indigo carmine (RDI-indigo) in assessing the invasion depth of colorectal tumors. **Methods:** Ninety images were obtained from 30 colorectal tumor cases using RDI-indigo, NBI, and MCE. Six endoscopists classified images using JNET classification for NBI, and pit pattern classification for RDI-indigo and MCE. JNET type 3 and pit pattern classification V irregular, high grade/V non-structure were correlated with pathological depth ≥pT1b. The primary outcome was the diagnostic accuracy for ≥pT1b. **Results:** Diagnostic accuracies for ≥pT1b were 85.0% (95% CI: 79.8–90.2%) for RDI-indigo, 79.4% (95% CI: 73.5–85.3%) for NBI, and 82.8% (95% CI: 77.3–88.3%) for MCE. Intraobserver agreement between RDI-indigo and MCE showed fair agreement (Cohen’s kappa = 0.39), while interobserver agreement was moderate for MCE (Fleiss’ kappa = 0.56) and fair for RDI-indigo (Fleiss’ kappa = 0.36). Gwet’s AC1 indicated substantial agreement across all assessments (0.69–0.80). **Conclusions:** As the first report evaluating RDI-indigo for colorectal tumors, this study suggests that RDI-indigo could serve as a complementary MCE-like tool for the diagnosis of ≥pT1b lesions.

## 1. Introduction

Among the risk factors of lymph node metastasis in T1 colorectal cancer, such as submucosal invasion ≥1000 µm (pT1b), lymphovascular invasion, poorly differentiated adenocarcinoma, signet-ring cell carcinoma or mucinous carcinoma component, and tumor budding grade 2/3, invasion depth is the only one that can be predicted preoperatively using endoscopic findings, making it crucial for determining the need for endoscopic or surgical resection.

Image-enhanced endoscopy enables differentiation of colorectal lesions and estimation of the invasion depth of colorectal carcinoma, and pit pattern diagnosis is recognized as one of the gold standards for determining the indication of endoscopic resection of colorectal lesions. However, magnifying chromoendoscopy with crystal violet staining (MCE), the dye used for pit pattern analysis, has several drawbacks, including potential carcinogenicity and prolonged procedure time [1].

The Japan NBI (narrow band imaging) Expert Team (JNET) classification, a standardized Japanese classification system for magnified NBI assessment of colorectal tumors proposed in 2014, enables prediction of the histological type and invasion depth of colorectal carcinoma [2]. In the JNET classification, types 2A and 3 have a positive predictive value of 90% for adenoma/pTis and pT1b lesions, respectively. Conversely, type 2B has a relatively lower positive predictive value of approximately 40–50% for pTis/pT1a carcinoma. This is because 12–21% of lesions diagnosed as type 2B include pT1b carcinomas [3,4,5]. Therefore, when a lesion is classified as JNET type 2B, additional diagnostic modalities such as pit pattern classification based on chromoendoscopy or endoscopic ultrasound should be used to comprehensively assess the invasion depth [6,7].

The EVIS X1 endoscopy system (Olympus Co., Tokyo, Japan), released in 2020, includes the red dichromatic imaging (RDI) mode, which enables image enhancement through simple scope manipulation alone without additional staining, and enhances contrast in deep blood vessels using narrow-band red, amber, and green light. This mode is particularly useful for identifying bleeding points during endoscopic treatment [8]. We observed that when RDI mode 1 was used after indigo carmine spraying, pit structures became visible in colorectal tumors. If the pit pattern classification used in MCE could be applied to diagnose lesions that are not indicated for endoscopic treatment, it may contribute to avoiding the risk of carcinogenesis and shortening the procedure time.

In this pilot study, we aimed to evaluate the diagnostic performance of RDI-indigo for the diagnosis of pT1b in colorectal tumors.

## 2. Materials and Methods

### 2.1. Study Design and Approval

This single-center retrospective observational study was designed as an exploratory investigation and was conducted in accordance with the Declaration of Helsinki and the ethical guidelines for medical and health research involving humans (Ministry of Health, Labour and Welfare, Japan). This study was approved by the institutional review board of The Jikei University School of Medicine (No. 37-014(12650)).

### 2.2. Patients and Data Collection

Patients aged ≥18 years who underwent endoscopic treatment and had pathologically confirmed lesions between 1 January 2024 and 31 December 2024 were included. Eligible cases comprised patients with colorectal tumors (including suspected lesions) that were indicated for endoscopic treatment based on endoscopic diagnosis and underwent magnification endoscopy using three modalities: RDI with indigo carmine spraying (RDI-indigo), NBI, and MCE. Cases were excluded if images were out of focus or had poor quality due to inadequate cleaning. This study was disclosed on our university’s website for opt-out notification, and patients who declined participation were excluded.

### 2.3. Endoscopic System and Setting

We used the EVIS X1 endoscopic system (Video System Center CV-1500; Olympus Corporation, Tokyo, Japan) and high-definition magnifying endoscopes, including the PCF-H290ZI (Olympus Corporation) and CF-XZ1200I (Olympus Corporation). To stabilize the focal distance during magnifying observations, a distal attachment hood was mounted on the tip of the endoscopes. The EVIS X1 system can promptly switch between image modalities (white light imaging, NBI, and RDI) via a button, and RDI mode 1 was utilized as the standard setting for RDI observation in this study.

### 2.4. Evaluation of Endoscopic Images

The region of interest was observed using magnifying endoscopy, and 30 lesions that were captured from the same endoscopic view using all three modalities (RDI-indigo, NBI, MCE) were selected and used as test images. An image catalog was created in which each colorectal tumor was represented by three images from different modalities. The images were randomly arranged based on a randomized table created using Excel software (Microsoft Corporation, Redmond, WA, USA).

All reviewers were instructed on how to apply and interpret the visibility scales by an organizer (T.F.), who was not an image reviewer in this study. The reviewers comprised three expert endoscopists and three trainees. Experts were board-certified by the Japan Gastroenterological Endoscopy Society (JGES) and were defined as having performed ≥1000 colonoscopies, whereas trainees were defined as those with an experience of <1000 colonoscopies. The reviewers classified NBI findings using the JNET classification, while RDI-indigo and MCE findings were classified using the pit pattern classification [9]. To evaluate the diagnostic performance, these classifications were correlated with pathological findings.

**Figure 1:**
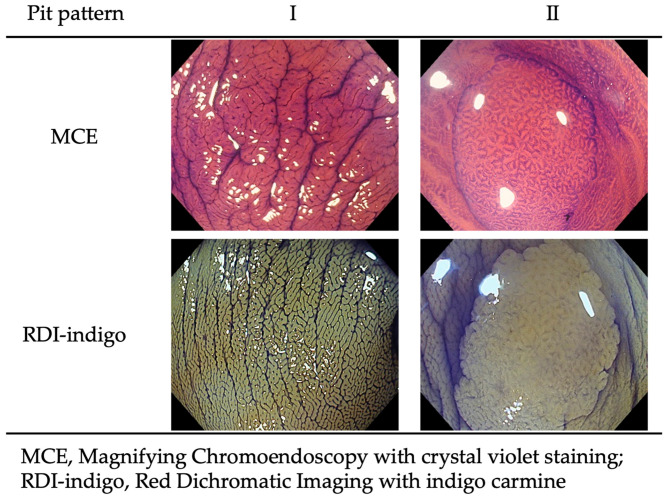
Representative images of pit patterns I and II using MCE and RDI-indigo. (**I**) Normal pits. (**II**) Stellate or papillary pits are identifiable in both modalities.

**Figure 2:**
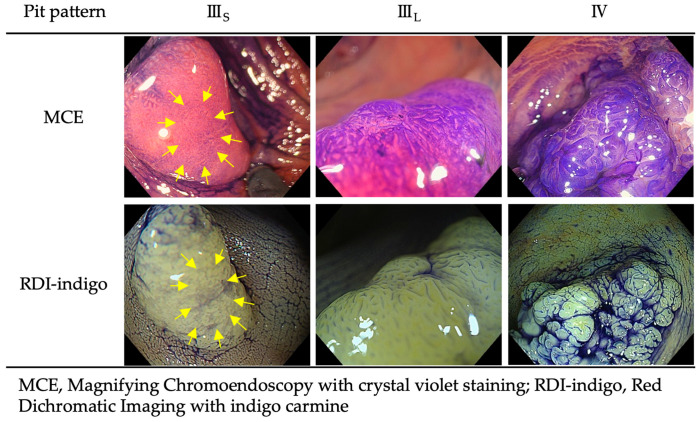
Representative images of pit patterns III and IV using MCE and RDI-indigo. (**III_S_**) Small round/tubular pits (indicated by arrows). RDI-indigo clearly visualizes indigo carmine pooling within the small pits. (**III_L_**) Large round/tubular pits. Indigo carmine pooling within large pits is observed. (**IV**) Branch-like or gyrus-like pits are observed in both modalities.

**Figure 3:**
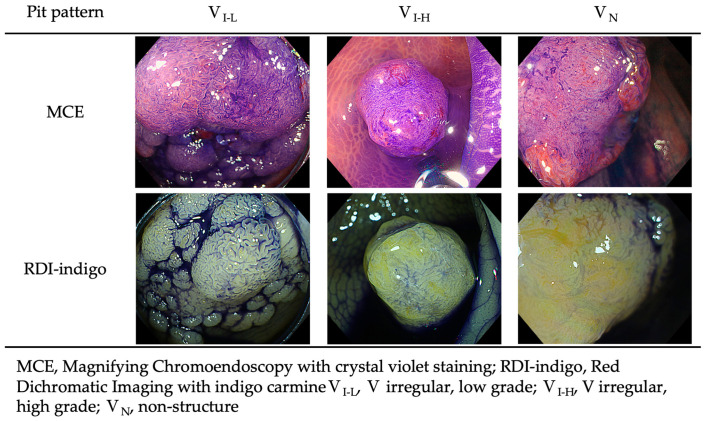
Representative images of pit patterns V_I-L_, V_I-H_, and V_N_ using MCE and RDI-indigo. (**V_I-L_**) Although pit irregularity is noted in RDI-indigo, the structure remains relatively preserved. (**V_I-H_**) The structure is obscure due to pit destruction, but some pits remain identifiable. (**V_N_**) Minimal indigo carmine pooling in non-structured areas with RDI-indigo.

JNET classification comprises four patterns (type 1, 2A, 2B, and 3) based on the magnified surface and vessel pattern. Type 1 corresponds to hyperplastic polyp/sessile Serrated lesion (with dysplasia) (SSL(D)), type 2A to adenoma, type 2B to carcinoma (intramucosal or submucosal invasion of less than 1000 µm; pTis/pT1a), and type 3 to carcinoma (deeper than pT1b; ≥pT1b). Pit pattern classification comprises eight patterns (I, II, III_S_, III_L_, IV, V irregular, low grade; V_I-L_, V irregular, high grade; V_I-H_, V non-structure; V_N_) based on the morphology and arrangement of colonic epithelial gland openings (pits). Pit patterns I and II correspond to hyperplastic polyp/SSL(D), III_S_, III_L_, and IV to adenoma, V_I-L_ to carcinoma (pTis/pT1a), and V_I-H_, V_N_ to carcinoma (≥pT1b). Representative images of pit pattern classification using MCE and RDI-indigo are shown in Figure 1, Figure 2 and Figure 3.

### 2.5. Outcome Measures

The primary outcome was the diagnostic accuracy for ≥pT1b among the three modalities: RDI-indigo, NBI, and MCE. The secondary outcomes were sensitivity, specificity, positive predictive value (PPV), and negative predictive value (NPV) for ≥pT1b for each modality.

### 2.6. Statistical Analysis

Continuous variables were presented as median and interquartile range (IQR: first quartile–third quartile). Categorical variables were summarized as frequencies and percentages. Intraobserver and inter-modality agreements were assessed using Cohen’s kappa coefficient, while interobserver agreement was assessed using Fleiss’ kappa coefficient. To complement these analyses, percent agreement and Gwet’s AC1 were calculated to account for potential prevalence imbalance. The calculated kappa values (both Cohen’s and Fleiss’ kappa) were interpreted according to the Landis and Koch criteria [10], and the values of <0.00, 0.00–0.20, 0.21–0.40, 0.41–0.60, 0.61–0.80, and 0.81–1.00 were considered to indicate poor, slight, fair, moderate, substantial, and almost perfect agreement, respectively. Accuracy, sensitivity, specificity, PPV, and NPV were expressed as point estimates with exact 95% confidence intervals (95% CIs). As this was a pilot study with a limited sample size, no formal hypothesis testing was performed, and results were interpreted descriptively to explore trends in diagnostic performance. All analyses were performed using Stata 14.0 (Stata Corp LP, College Station, TX, USA).

## 3. Results

### 3.1. Patients and Lesion Characteristics

**Figure 4:**
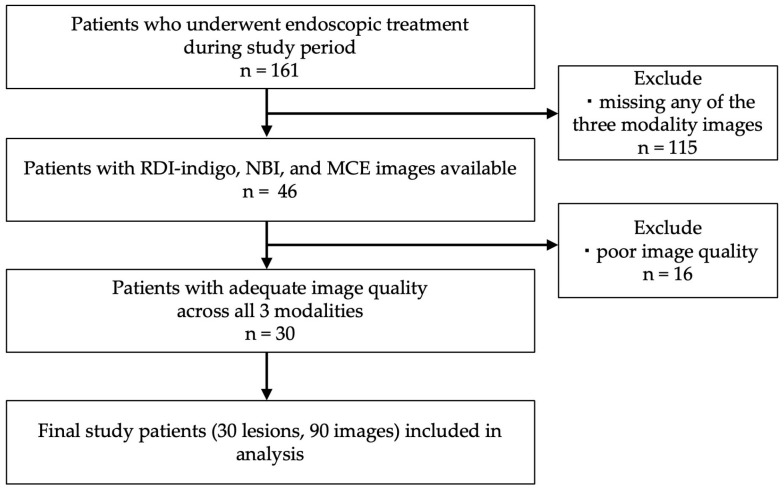
A flow diagram. RDI-indigo, red dichromatic imaging with indigo carmine spraying; NBI, narrow band imaging; MCE, magnifying chromo-endoscopy with crystal violet staining.

Of the 161 initial patients, 115 were excluded due to missing imaging modalities (RDI-indigo, NBI, and MCE) and 16 due to poor image quality. Finally, 30 patients (30 colorectal lesions, represented by 90 images) were classified by six reviewers, and the resulting evaluation data were included in the analysis (Figure 4).

The median lesion size was 24.5 mm (IQR: 20.0–44.5 mm). The distribution of lesion sizes was as follows: 5 mm or smaller (n = 0), 6–10 mm (n = 1), 11–15 mm (n = 2), 16–20 mm (n = 10), and 21 mm or larger (n = 17). The lesions were located in the cecum (n = 5), ascending colon (n = 6), transverse colon (n = 5), descending colon (n = 1), sigmoid colon (n = 5), and rectum (n = 8). According to macroscopic morphology, 13 lesions were protruding, 3 were flat elevated, and 14 were depressed. Histopathological examination identified 3 hyperplastic polyps/SSL(D), 5 adenomas, 17 well-differentiated adenocarcinomas, 4 moderately differentiated adenocarcinomas, and 1 mucinous adenocarcinoma. Among the 22 cancerous lesions, the histological invasion depth was classified as follows: pTis (n = 14), pT1a (n = 1), pT1b (n = 6), and pT4a (n = 1). Lymphatic invasion (Ly) was absent in 19 cases (Ly0) and present in 3 cases (Ly1). Vascular invasion (V) was absent in 19 cases (V0) and present in 3 cases (V1). These data are summarized in Table 1.

### 3.2. Accuracy of Each Modality for ≥pT1b

The diagnostic accuracy of RDI-indigo, NBI, and MCE for ≥pT1b, the primary outcome of this study, is presented in Table 2.

### 3.3. Diagnostic Performance of Each Modality for ≥pT1b

The diagnostic performance of RDI-indigo, NBI, and MCE for ≥pT1b, assessed by sensitivity, specificity, PPV, and NPV, is presented in Table 3.

Supplementary Table S1 shows the diagnostic performance for each modality, including all lesions (based on 180 total evaluations).

### 3.4. Diagnostic Performance of Each Modality for ≥pT1b Classified as JNET Type 2B

Since JNET type 2B lesions represent the most clinically critical group for treatment decision-making, a subgroup analysis of lesions classified as JNET type 2B by at least one reviewer is presented in Table 4.

### 3.5. Intrarobserver Agreement

Intraobserver agreement between RDI-indigo and MCE for the diagnosis of V_I-H_ and V_N_ was assessed for each of the six reviewers. Overall, the percent agreement was acceptable, with Cohen’s kappa indicating fair agreement and Gwet’s AC1 indicating substantial agreement. Agreement varied among individual reviewers, with Cohen’s kappa ranging from fair to moderate. The wide CIs observed for individual reviewers likely reflect the limited sample size. Detailed data for each reviewer is shown in Table 5.

### 3.6. Interobserver Agreement

Interobserver agreement among the six reviewers was evaluated for MCE and RDI-indigo separately. For MCE, Fleiss’ kappa indicated moderate agreement, with percent agreement and Gwet’s AC1 both suggesting substantial agreement. For RDI-indigo, Fleiss’ kappa suggested fair agreement; however, percent agreement and Gwet’s AC1 both indicated substantial agreement. The discrepancy between Fleiss’ kappa and the other measures for RDI-indigo likely reflects a prevalence imbalance between the two diagnostic categories, a known limitation of the kappa statistic. The interobserver agreement for MCE and RDI-indigo among the six reviewers is presented in Table 6.

## 4. Discussion

RDI uses three narrow-band lights—green (520–550 nm), amber (595–610 nm), and red (620–640 nm)—selected from five LEDs. These wavelengths are combined using dichroic filters, and the spectrum is further narrowed by an RDI filter to generate light suitable for RDI observation. Based on the difference in attenuation between reflected light at 595–610 nm and 620–640 nm, RDI enhances visualization of thick blood vessels. RDI offers three observation modes. As indigo carmine absorbs light at 595–610 nm and 620–640 nm more strongly than that at 520–550 nm, it appears “deep blue” in RDI mode 1 or 2 [11]. RDI-indigo imaging visualized structures formed by indigo carmine pooling in the pits; therefore, uniform spraying of indigo carmine is considered essential.

In this pilot study, the diagnostic accuracy for ≥pT1b was 85.0% with RDI-indigo, 79.4% with NBI, and 82.8% with MCE. These results are consistent with previously reported data. The diagnostic accuracy of MCE for deep submucosal invasion has been reported to range from 85% to 99% [12,13,14], while that of magnifying NBI has been reported to range from approximately 85% to 93% [15,16,17]. RDI-indigo demonstrated comparable diagnostic accuracy to previously reported values.

When comparing diagnostic performance based on the overlap of 95% CIs, the overall diagnostic accuracy showed substantial overlap among all three modalities, indicating comparable performance. However, for sensitivity, the 95% CI of MCE (55.4–84.3%) did not overlap with that of NBI (10.3–36.8%), suggesting a clear superiority of MCE over NBI in detecting ≥pT1b lesions. RDI-indigo showed intermediate sensitivity (95% CI: 32.0–63.6%), with CIs overlapping both MCE and NBI. Regarding specificity, RDI-indigo (91.7–98.8%) and NBI (92.7–99.2%) demonstrated higher specificity than MCE (79.3–91.5%), with no CI overlap. Despite these statistical overlaps for RDI-indigo, from a clinical perspective, the lower point estimates of sensitivity for both RDI-indigo (47.6%) and NBI (21.4%)—compared to 71.4% for MCE—warrant careful consideration. A false-negative assessment may result in endoscopic treatment being performed for lesions that should be treated surgically. Therefore, when a lesion is diagnosed as pT1a by RDI-indigo or NBI, confirming the assessment with MCE may be a prudent approach to minimize the risk of underdiagnosis.

Importantly, this concern regarding sensitivity was partially addressed in the subgroup analysis restricted to JNET type 2B lesions. In this subgroup, diagnostic accuracy was comparable between RDI-indigo (78.6%) and MCE (82.1%). The sensitivity of RDI-indigo improved from 47.6% in the full cohort to 81.3% in the JNET type 2B subgroup, while that of MCE remained similar (71.4% vs. 72.4%). The 95% CIs for the sensitivity of RDI-indigo (54.4–96.0%) and MCE (52.8–87.3%) showed substantial overlap, suggesting comparable sensitivity between the two modalities in this subgroup. Regarding specificity, RDI-indigo decreased from 96.4% to 77.9%, whereas MCE remained largely unchanged (86.2% vs. 87.3%). These findings suggest that RDI-indigo may be particularly effective for JNET type 2B lesions, where the distinction between pT1a and ≥pT1b is most critical for treatment decision-making.

Intraobserver and interobserver agreement for the diagnosis of ≥pT1b showed fair-to-moderate kappa values (Cohen’s kappa = 0.39; Fleiss’ kappa = 0.36–0.56); however, percent agreement was relatively high at 79.4% and 82.2–84.7%. This discrepancy may reflect the kappa paradox, whereby prevalence imbalance artificially deflates kappa coefficients [18]. Gwet’s AC1, a prevalence-robust alternative, consistently indicated substantial agreement across all assessments (intraobserver: Gwet’s AC1 = 0.69; interobserver MCE: Gwet’s AC1 = 0.70; interobserver RDI-indigo: Gwet’s AC1 = 0.80), suggesting that the relatively low kappa values reflect prevalence imbalance rather than true diagnostic discordance.

Taken together, RDI-indigo may have a potential role in supporting the diagnosis of ≥pT1b in colorectal tumors. However, the appearance of pit patterns may vary depending on the method of indigo carmine application, and interpretation remains inherently subjective. RDI-indigo should currently be considered a complementary tool, serving as a simplified, MCE-like method for cases in which NBI observation yields a diagnosis of JNET type 2B, rather than a standalone diagnostic modality.

Although diagnosis using NBI was performed based on the JNET classification, which is widely adopted in Japan, the NBI International Colorectal Endoscopic (NICE) classification—a classification that evaluates lesion color, vascular pattern, and surface pattern using NBI regardless of magnification—is more widely accepted worldwide [19,20,21]. NICE type 3 corresponds to JNET type 3, and Hayashi et al. reported that the accuracy for diagnosing deep submucosal invasion in high-confidence predictions by experts was 84.1%. Future comparisons incorporating the NICE classification, which includes both magnifying and non-magnifying NBI images, would be of interest.

Moreover, the application of artificial intelligence (AI) in gastrointestinal endoscopy has expanded rapidly in recent years, demonstrating remarkable progress in lesion detection and characterization. However, their clinical effectiveness in real-world settings remains debated [22], and diagnostic performance in predicting invasion depth continues to pose a challenge. Notably, Minami et al. reported that the accuracy of a convolutional neural network in diagnosing deep submucosal invasive colorectal cancer was 74.4% [23], indicating that further technical improvements are warranted. At present, detailed optical evaluation using image-enhanced endoscopy, such as MCE and RDI-indigo, will continue to play an indispensable complementary role in making critical treatment decisions.

This study has some limitations. First, the small sample size reduced statistical power, thereby limiting the ability to draw definitive conclusions. Second, a selection bias in pathological findings cannot be excluded, and the present study should be regarded as a descriptive, exploratory investigation. Third, multiple evaluations of the same lesion may not have been statistically independent, potentially leading to an underestimation of the standard error and overly narrow CIs. Fourth, as this study was based on retrospective still-image assessment, the findings may not reflect real-time diagnostic performance in real-world clinical practice. Fifth, since the eligible cases were confined to lesions that underwent endoscopic treatment, one case of advanced protruding cancer (pT4a), classified within the ≥pT1b group, was inevitably included. This may have influenced the diagnostic performance results. These limitations should be addressed in future prospective studies informed by the results of this pilot study.

## 5. Conclusions

As the first report evaluating RDI-indigo for colorectal tumors, this study suggests that RDI-indigo could serve as a simplified MCE-like complementary imaging approach for the diagnosis of ≥pT1b lesions, particularly in lesions classified as JNET Type 2B by NBI. Further prospective studies with larger sample sizes are warranted to validate these findings.

## Figures and Tables

**Table 1:** Lesion characteristics.

		n = 30		n = 30
Size, mm (%)	−5	0		
	6–10	1 (3.3)		
	11–15	2 (6.7)		
	16–20	10 (33.3)		
	21-	17 (56.7)		
	Median (IQR)	24.5 (20.0–44.5)		
Location, n (%)	Cecum	5 (16.7)	Right colon *	16 (53.3)
	Ascending colon	6 (20.0)		
	Transverse colon	5 (16.7)		
	Descending colon	1 (3.3)	Left colon **	6 (20.0)
	Sigmoid colon	5 (16.7)		
	Rectum	8 (26.7)		
Morphology, n (%)	Protruding	13 (43.3)		
	Flat elevated	3 (10.0)		
	Depressed †	14 (46.7)		
Histological diagnosis, n (%)	Hyperplastic polyp/SSL(D)	3 (10.0)		
	Adenoma	5 (16.7)		
	Well-differentiated adenocarcinoma	17 (56.7)		
	Moderately differentiated adenocarcinoma	4 (13.3)		
	Mucinous adenocarcinoma	1 (3.3)		
Histological depth, n (%)	pTis	14 (46.7)		
	pT1a	1 (3.3)		
	pT1b	6 (20.0)		
	pT4a	1 (3.3)		
Ly, n (%) (n = 22) ††	0	19/22 (86.4)		
	1	3/22 (13.6)		
V, n (%) (n = 22) ††	0	19/22 (86.4)		
	1	3/22 (13.6)		

* Cecum-transverse colon. ** Descending-sigmoid colon. † IIc, IIa+IIc, Is+IIc. †† Excluding 8 non-cancerous lesions.

**Table 2:** Accuracy of each modality for ≥pT1b.

Modality	Accuracy (95% CI)
RDI-indigo (V_I-H_, V_N_)	85.0% (79.8–90.2%)
NBI (Type 3)	79.4% (73.5–85.3%)
MCE (V_I-H_, V_N_)	82.8% (77.3–88.3%)

RDI-indigo, red dichromatic imaging with indigo carmine spraying; NBI, narrow band imaging; MCE, magnifying chromoendoscopy with crystal violet staining; V_I-H _, V irregular, high grade; V_N_, V non-structure.

**Table 3:** Diagnostic performance of each modality for the diagnosis of ≥pT1b.

	Sensitivity (95% CI)	Specificity (95% CI)	PPV (95% CI)	NPV (95% CI)
RDI-indigo (V_I-H_, V_N_)	47.6% (32.0–63.6%)	96.4% (91.7–98.8%)	80.0% (59.3–93.2%)	85.8% (79.3–90.9%)
NBI (Type 3)	21.4% (10.3–36.8%)	97.1% (92.7–99.2%)	69.2% (38.6–90.9%)	80.2% (73.4–86.0%)
MCE (V_I-H_, V_N_)	71.4% (55.4–84.3%)	86.2% (79.3–91.5%)	61.2% (46.2–74.8%)	90.8% (84.5–95.2%)

RDI-indigo, red dichromatic imaging with indigo carmine spraying; NBI, narrow band imaging; MCE, magnifying chromoendoscopy with crystal violet staining; V_I-H _, V irregular, high grade; V_N_, V non-structure.

**Table 4:** Diagnostic performance for ≥pT1b classified as JNET type 2B by at least one reviewer.

	Accuracy (95% CI)	Sensitivity (95% CI)	Specificity (95% CI)	PPV (95% CI)	NPV (95% CI)
RDI-indigo (V_I-H_, V_N_)	78.6% (70.0–87.3%)	81.3% (54.4–96.0%)	77.9% (66.2–87.1%)	46.4% (27.5–66.1%)	94.6% (85.1–98.6%)
MCE (V_I-H_, V_N_)	82.1% (74.0–90.3%)	72.4% (52.8–87.3%)	87.3% (75.5–94.7%)	75.0% (55.1–89.3%)	85.7% (73.8–93.6%)

RDI, red dichromatic imaging; NBI, narrow band imaging; MCE, magnifying chromoendoscopy with crystal violet staining.

**Table 5:** Intraobserver agreement between RDI-indigo and MCE for each of six reviewers for the diagnosis of ≥pT1b.

Reviewer	All	A	B	C	D	E	F
Cohen’s kappa value (95% CI)	0.39 (0.26–0.53)	0.26 (0.09–0.61)	0.45 (0.11–0.79)	0.24 (0.01–0.48)	0.60 (0.27–0.93)	0.22 (0.07–0.51)	0.45 (0.12–0.79)
Percent agreement % (95% CI)	79.4 (73.0–84.7)	73.3 (55.6–85.1)	83.3 (66.4–92.7)	83.3 (66.4–92.7)	83.3 (66.4–92.7)	73.3 (55.6–85.8)	80.0 (62.7–90.5)
Gwet’s AC1 (95% CI)	0.69 (0.59–0.80)	0.72 (0.53–0.89)	0.76 (0.53–0.99)	0.79 (0.58–1.00)	0.72 (0.25–0.94)	0.61 (0.31–0.91)	0.69 (0.42–0.85)

**Table 6:** Interobserver agreement for RDI-indigo and MCE among the six reviewers for the diagnosis of ≥pT1b.

	RDI-Indigo	MCE
Fleiss’ kappa value (95% CI)	0.36 (0.16–0.55)	0.56 (0.36–0.75)
Percent agreement % (95% CI)	84.7 (76.1–93.2)	82.2 (74.6–90.0)
Gwet’s AC1 (95% CI)	0.80 (0.66–0.94)	0.70 (0.55–0.86)

## Data Availability

The data that support the findings of this study are available from the corresponding author, T.F., upon reasonable request.

## Supplementary Materials

The following supporting information can be downloaded at https://www.mdpi.com/article/10.3390/diagnostics16111739/s1: Table S1: Diagnostic performance for all lesions, adenoma, pTis/pT1a of each modality based on 180 evaluations.

## Abbreviations

The following abbreviations are used in this manuscript:

NBI	Narrow Band Imaging
JNET	Japan NBI Expert Team
MCE	Magnifying Chromoendoscopy with Crystal Violet Staining
RDI	Red Dichromatic Imaging
RDI-indigo	Red Dichromatic Imaging with Indigo Carmine
T1b	Depth of Submucosal Invasion ≥1000 µm
CIs	Confidence Intervals
V_I-L_	V Irregular, Low Grade
V_I-H_	V Irregular, High Grade
V_N_	V Non-structure
SSL(D)	Sessile Serrated Lesion (with Dysplasia)
PPV	Positive Predictive Value
NPV	Negative Predictive Value
IQR	Interquartile Range
