# Supplementary material for: Assessment of Red Dichromatic Imaging with Indigo Carmine for Identifying Deep Submucosal Invasion in Colorectal Tumors: A Pilot Study
Source: Diagnostics (Basel). 2026 Jun 5;16(11):1739. doi: 10.3390/diagnostics16111739 (PMC13257305; doi:10.3390/diagnostics16111739)
Supplement: Supplementary file 1 [file diagnostics-16-01739-s001.zip › diagnostics-4325697-supplementary.pdf]

Supplementary Table S1. Diagnostic performance for all lesions, adenoma, pTis/pT1a of each modality based on 180 evaluations.

	Sensitivity	Specificity	PPV	NPV	Accuracy
RDI-indigo (All lesions)	42.9% (35.3–50.7%)	75.5% (70.8–79.8%)	44.2% (36.4–52.1%)	74.5% (69.8–78.9%)	65.3% (61.–69.4%)
RDI-indigo（Ⅲ$_S$，Ⅲ$_L$，Ⅳ)	73.3% (54.1–87.7%)	55.3% (47.0–63.4%)	24.7% (16.2–35.0%)	91.2% (83.4–96.1%)	58.3% (51.1–65.5%)
RDI-indigo (Ⅴ$_{I\text{-}L}$)	31.3% (22.2–41.5%)	77.4% (67.0–85.8%)	61.2% (46.2–74.8%)	49.6% (40.8–58.5%)	52.8% (45.5–60.1%)
NBI (All lesions)	50.4% (41.5–59.3%)	75.7% (71.2–79.7%)	39.4% (31.9–47.3%)	82.9% (78.8–86.6%)	69.6% (65.8–73.5%)
NBI (Type 2A)	50.0% (31.3–68.7%)	64.7% (56.5–72.3%)	22.1% (12.9–33.8%)	86.6% (78.9–92.3%)	62.2% (55.1–69.3%)
NBI (Type 2B)	71.9% (58.5–83.0%)	65.0% (55.9–73.4%)	48.8% (37.7–60.0%)	83.3% (74.4–90.2%)	67.7% (60.4–74.1%)
MCE (All lesions)	57.7% (49.9–65.3%)	81.5% (77.1–85.3%)	58.4% (50.5–66.0%)	81.0% (76.7–84.9%)	74.1% (70.4–77.8%)
MCE（Ⅲ$_S$，Ⅲ$_L$，Ⅳ)	20.0% (7.7–38.6%)	84.0% (77.1–89.5%)	20.0% (7.7–38.6%)	84.0% (77.1–89.5%)	73.3% (66.9–79.8%)
MCE (Ⅴ$_{I\text{-}L}$)	63.5% (53.1–73.1%)	69.0% (58.0–78.7%)	70.1% (59.4–79.5%)	62.4% (51.7–72.2%)	66.1% (59.2–73.0%)

RDI-indigo, red dichromatic imaging with indigo carmine spraying; NBI, narrow band imaging; MCE, magnifying chromoendoscopy with crystal violet staining; V$_{I\text{-}L}$, V irregular, low grade; PPV, positive predictive value; NPV, negative predictive value.
